# Supplementary figures and images for: Unraveling the Heterogeneous Mutational Signature of Spontaneously Developing Tumors in MLH1−/− Mice
Source: Cancers (Basel). 2019 Oct 2;11(10):1485. doi: 10.3390/cancers11101485 (PMC6827043; doi:10.3390/cancers11101485)

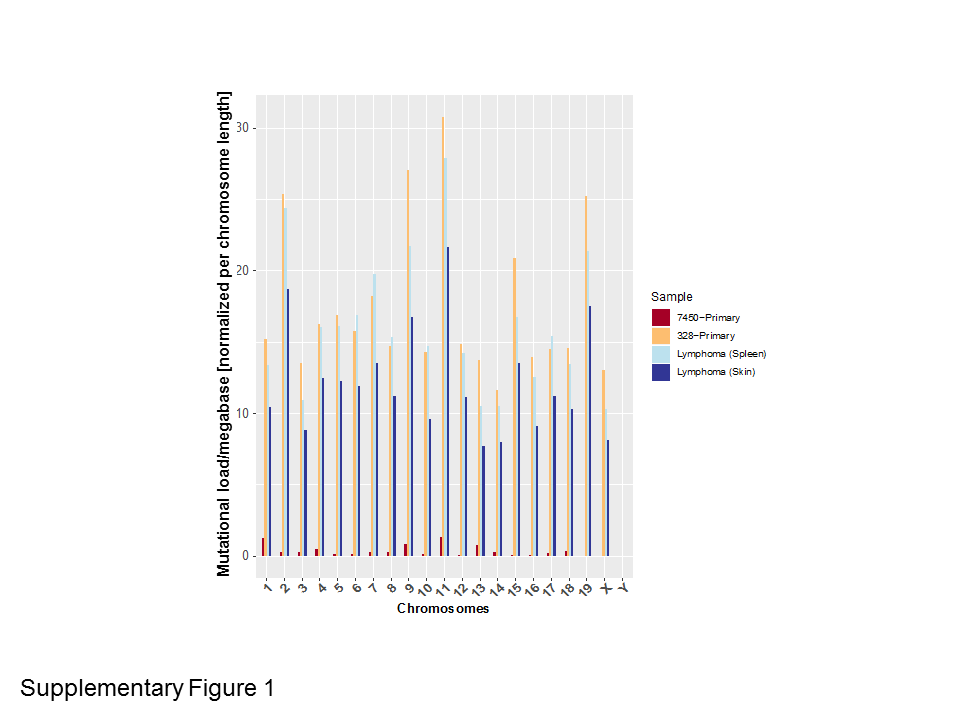

Supplement: Supplementary file 1 [file cancers-11-01485-s001.zip › supplementary figures/Folie1.TIF]

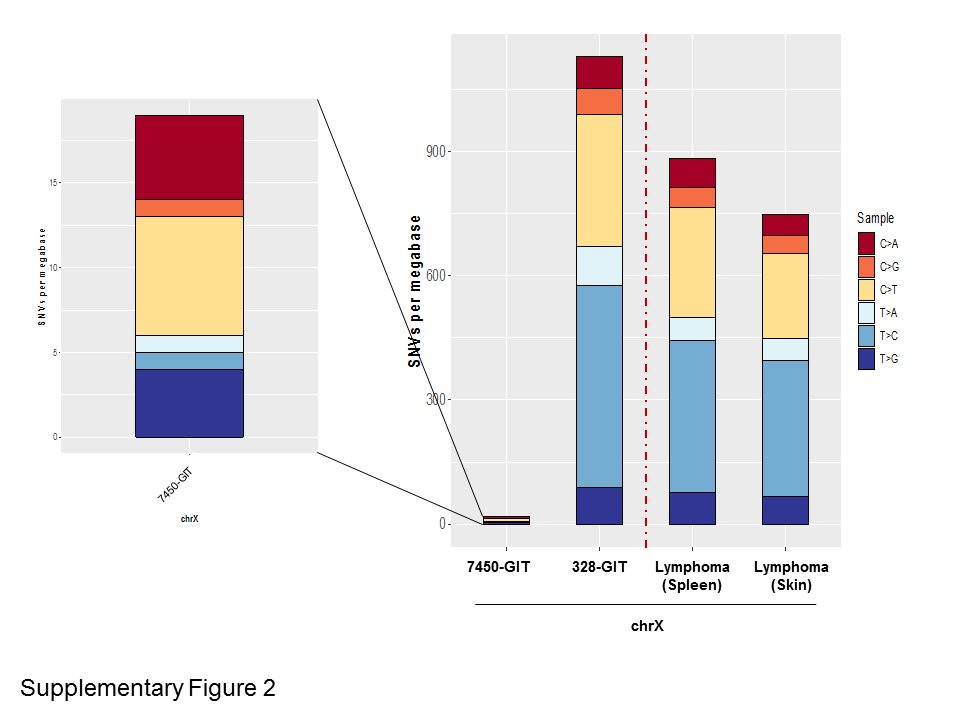

Supplement: Supplementary file 1 [file cancers-11-01485-s001.zip › supplementary figures/Folie2.TIF]
